# Norbornadiene Quadricyclane as Multimode Photoswitches: Synergistic Light and Protonation‐Controlled Heat Release

**DOI:** 10.1002/cssc.202501005

**Published:** 2025-08-19

**Authors:** Adil S. Aslam, Monika Shamsabadi, Rebecca J. Salthouse, Joakim Andréasson, Kasper Moth‐Poulsen

**Affiliations:** ^1^ Department of Chemistry and Chemical Engineering Chalmers University of Technology 41296 Gothenburg Sweden; ^2^ Department of Chemical Engineering Universitat Politècnica de Catalunya (EEBE) Eduard Maristany 10–14 08019 Barcelona Spain; ^3^ The Institute of Materials Science of Barcelona ICMAB‐CSIC Bellaterra 08193 Barcelona Spain; ^4^ Catalan Institution for Research & Advanced Studies (ICREA) Pg. Lluís Companys 23 08010 Barcelona Spain

**Keywords:** molecular photoswitches, norbornadienes, protonation, pyridine, solar energy storage

## Abstract

Two low molecular weight acceptor–acceptor norbornadiene (NBD) photoswitches functionalized with *meta*‐ and *ortho*‐substituted pyridine and cyano groups are presented. These molecular systems can be converted between four states in response to light, acid, base, and heat. Quantitative conversion to higher energy metastable quadricyclane (QC) photoisomers is achieved upon UV irradiation, with photoisomerization quantum yields of 37% and 24% for **NBD 1** and **2**. The thermal half‐lives, *t*_1/2_, of 70 and 205 days greatly surpass those of previously reported pyridine‐functionalized norbornadiene switches, as well as other acceptor–acceptor systems. In particular, the *ortho*‐positioning of the pyridine has a profound effect on the half‐life; **QC 2** in its unprotonated form has the longest *t*_1/2_ of 205 days, while protonation to **QCH** ^**+**^ **2** completely hinders the thermal back conversion, allowing energy to be stored in this state indefinitely. The stored energy can then be released as required upon the addition of a base, followed by thermal reversion to **NBD 2**. The energy storage densities are 162 and 393 kJ kg^−1^ for **NBD 1** and **NBD 2**, respectively. This approach of multimodal photoswitches can be applied to molecular solar thermal energy storage devices to enable long‐term energy storage and on‐demand controlled release.

## Graphical abstract

Norbornadiene is conjugated with cyano and pyridine acceptor groups. The molecules represent a class of low molecular weight norbornadiene–quadricyclane multimode switches with protonation/deprotonation and molecular solar thermal energy storage properties (see graphic).

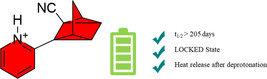

## 1 Introduction

**Figure 1:**
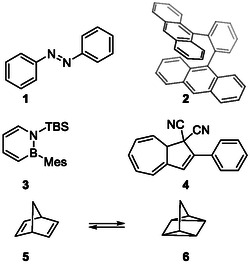
Commonly studied photoswitches for MOST technology.

Artificial molecular systems with on‐demand functionalities have the potential to drive advancements in sustainable energy technologies. Molecular photoswitches, a rapidly expanding field, offer promising solutions for solar energy conversion and storage. Their ability to undergo reversible isomerization induced by light and thermal reactions allows for the absorption, storage, and controlled release of solar energy as heat. This strategy, known as molecular solar‐thermal energy storage (MOST) or solar thermal fuels,^[^ ^1^ ^]^ encompasses various organic photoswitches (**Figure** **1**), including azobenzene **(1** **)**,^[^ ^2^, ^3^, ^4^ ^]^ anthracene systems **(2** **)**,^[^ ^5^ ^]^ azaborinines **(3** **)**,^[^ ^6^ ^]^ dihydroazulene (DHA) **(** **4** **)**,^[^ ^7^ ^]^ and the norbornadiene (NBD)/quadricyclane (QC) couple **(5/** **6** **)**.^[^ ^8^ ^]^

Among these systems, the NBD/QC pair has garnered significant attention due to its potential for device integration,^[^ ^9^ ^]^ high energy density,^[^ ^10^ ^]^ and extended thermal storage, with reported half‐lives of up to 18 years.^[^ ^11^ ^]^ However, a key limitation of NBD‐based systems is that their absorption lies predominantly in the UV region, necessitating molecular modifications to improve their efficiency in practical applications. One approach to utilize UV‐absorbing molecules is indirect photoisomerization through triplet sensitization.^[^ ^12^ ^]^ Another approach involves functionalizing NBD with donor/acceptor or acceptor/acceptor units to redshift the absorption,^[^ ^13^, ^14^, ^15^, ^16^ ^]^ while other strategies explore dimeric or oligomeric architectures.^[^ ^17^ ^]^ However, these often lead to increased molar mass, reducing energy density.^[^ ^18^ ^]^

Our group previously introduced cyano groups as compact acceptor units to address these challenges, providing a pathway toward low molecular weight NBDs with redshifted absorption.^[^ ^19^ ^]^ While donor/acceptor‐functionalized NBDs have been widely explored, acceptor/acceptor systems remain underrepresented. Dicyano‐functionalized norbornadienes were first investigated by Yoshida and colleagues in the 1980s, showing promise as acceptor/acceptor systems with a bathochromic shift in absorption. However, at the time, donor/acceptor systems were considered more favorable for energy storage, which rendered dicyano‐NBDs in MOST applications underexplored, despite their potential.^[^ ^20^ ^]^ In 1988, 2,3‐dipyridylnorbornadienes **NBD** **(7)** were reported and these systems demonstrated redshifted absorption in acidic media (up to 400 nm), but they were not evaluated for MOST applications.^[^ ^21^ ^]^ Pyridine adds extra functionality to the system with the potential of pH‐controlled photoswitching, a strategy that has been employed by Nielsen et al. for a DHA/vinylheptafulvene (VHF) framework conjugated with aniline and pyridine units.^[^ ^22^, ^23^ ^]^ More recently, an orthogonal photoswitching approach was introduced where pyridyl‐functionalized **NBD** **(8)** chelated metal ions, forming a four‐state system with red‐shifted absorptions for the chelate complexes as well as shorter half‐lives, mimicking electrochemically triggered cycloreversion.^[^ ^24^ ^]^ However, these existing systems face several drawbacks: 1) higher molar mass (246–508 g mol^−1^), 2) incomplete photoconversion (photostationary states of 37%–90% NBD), and 3) short thermal storage durations (half‐lives of 63–402 min).

**Figure 2:**
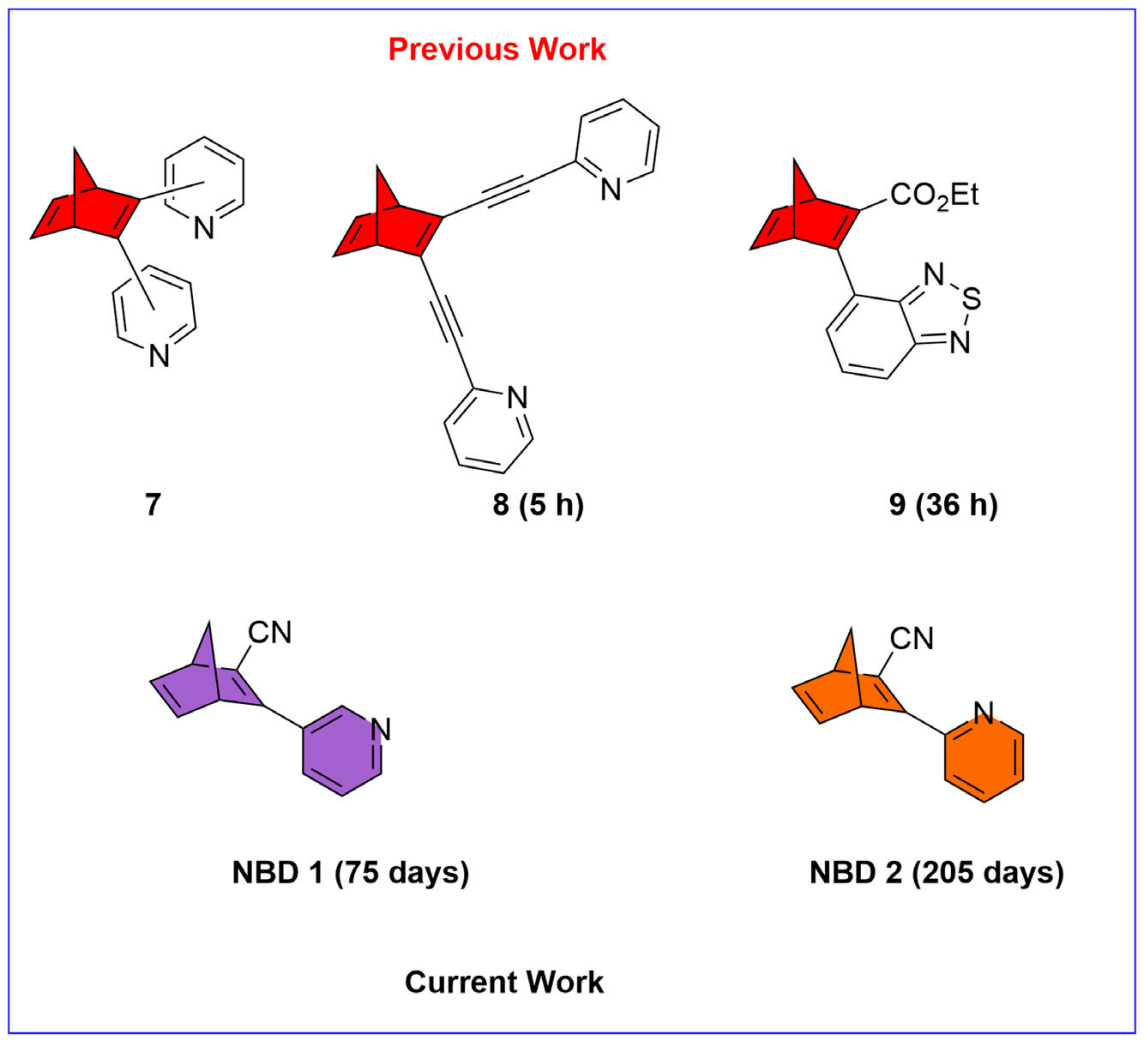
Representation of previous and current work with half‐lives in parentheses.

Given our work on acceptor/acceptor systems **NBD** **(** **9** **)**,^[^ ^15^ ^]^ molecular logic gates,^[^ ^25^ ^]^ and previous work on pH‐controlled photoswitching by Nielsen et al.,^[^ ^22^, ^23^ ^]^ we wanted to explore the substitution of norbornadiene with pyridine and cyano units. In this work, we report the synthesis of low molecular weight acceptor/acceptor NBDs with pyridine and cyano units directly linked to the NBD core, i.e., with no ethynyl linkers (**NBD 1** and **NBD 2**), designed to achieve extended thermal half‐lives, and multimodal energy storage capabilities owing to the pyridine functionality. These systems provide insight into structure–property relationships governing MOST performance. Our findings contribute to the advancement of molecular photoswitches for solar thermal energy storage applications (**Figure** **2**).

## 2 Results and Discussion

### 2.1 Synthesis

**Scheme 1:**
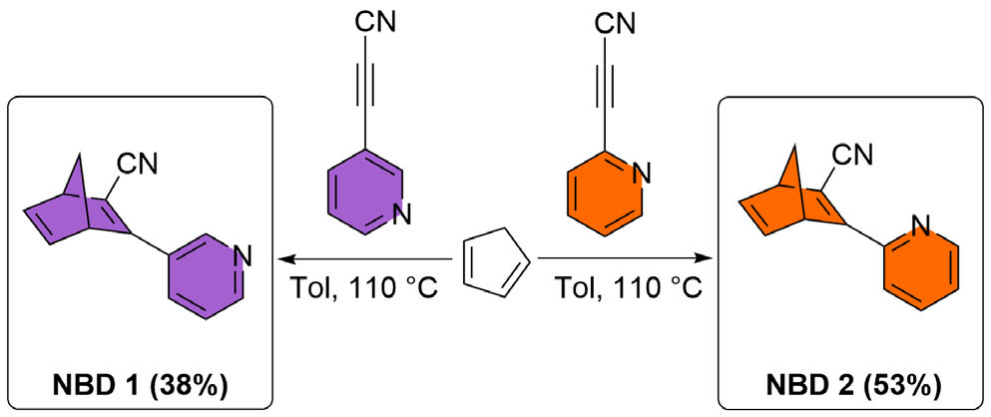
Synthetic route to **NBD 1** and **NBD 2** through a Diels–Alder reaction between the respective alkyne and cracked cyclopentadiene.

In the first step, 2‐ or 3‐iodo‐pyridine was coupled with propargyl alcohol via a Sonogashira coupling. The resulting pyridine‐propargyl alcohol was then oxidized to the nitrile using a refined method.^[^ ^26^ ^]^ Finally, the acetylene nitrile underwent a Diels–Alder reaction with freshly cracked cyclopentadiene, yielding **NBD 1** and **NBD 2** in moderate yields (**Scheme** **1**). Both the cyano and pyridine substituents reduce the electron density of the π‐system, thereby enhancing dienophilicity and facilitating the Diels–Alder cycloaddition, with the cyano group exerting a stronger effect than pyridine.

### 2.2 Photoswitching

**Figure 3:**
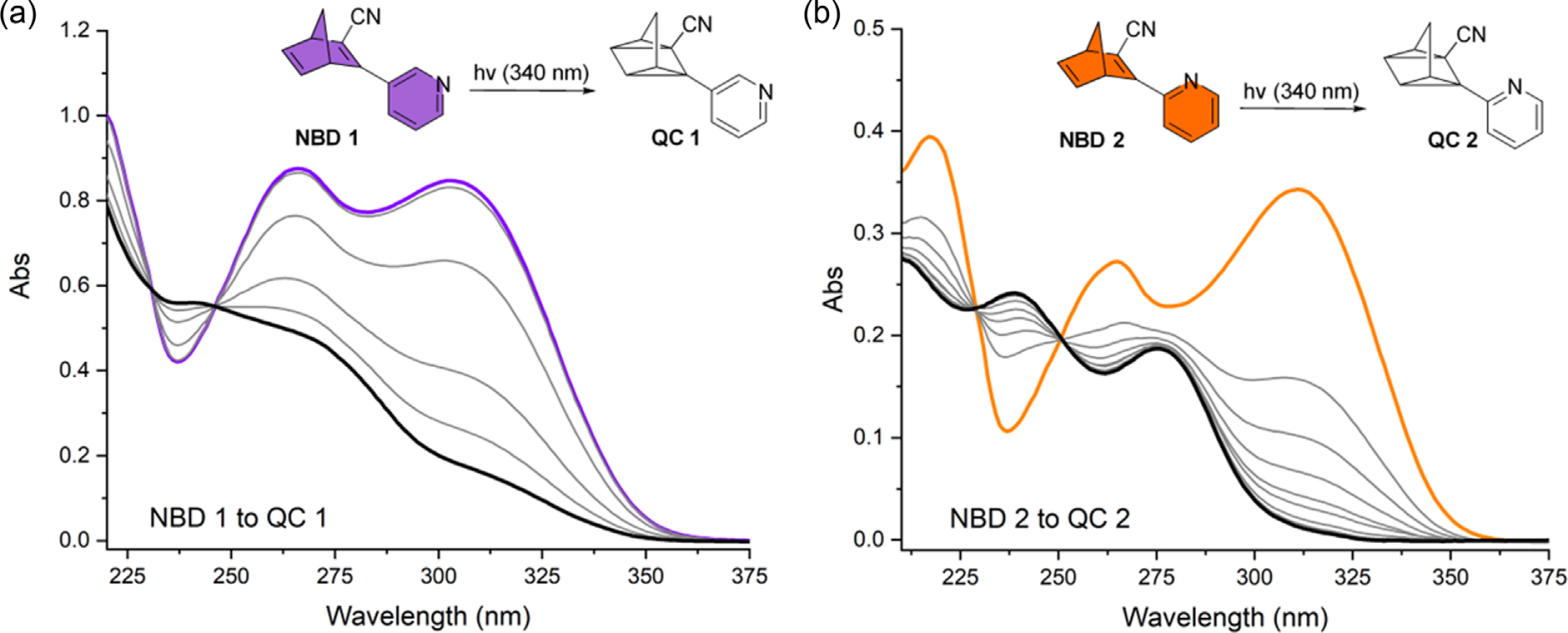
UV‐Vis spectra of NBD‐to‐QC conversion recorded in acetonitrile (acetonitrile was used for UV‐Vis spectroscopy due to its excellent transparency in the UV region) upon LED irradiation (340 nm, 3220 mW): a) a decrease in the absorbance due to photoisomerization of **NBD 1** (thick purple) to **QC 1** (thick black) after 30 min (5 min intervals between each spectrum) of irradiation; b) a decrease in the absorbance due to photoisomerization of **NBD 2** (thick orange) to **QC 2** (thick black) after 30 min of irradiation (5 min intervals between each spectrum).

The UV‐Vis absorption spectra of **NBD 1** and **NBD 2** in acetonitrile are shown in **Figure** **3** (purple and orange spectra, respectively), the absorption onsets, absorption maxima, and molar extinction coefficients are listed in **Table** **1** and **2**. **NBD 1** and **NBD 2** exhibit onset wavelengths, *λ*_ons_, of 360 and 362 nm and absorption maxima, *λ*_max_, of 308 and 311 nm, respectively. These values are blue‐shifted in comparison to the dipyridyl‐substituted **NBD** **(8)**, with *λ*_ons_ = 436 nm and *λ*_max_ = 355 nm,^[^ ^24^ ^]^ likely due to the decreased conjugation pathway owing to the smaller structures of the NBDs reported here. Irradiation with a 340 nm LED induced photoisomerization of NBD to QC, disrupting conjugation between the norbornadiene core and pyridine unit (Figure 3). Isosbestic points at 231 and 246 nm for **NBD 1** and at 229 and 250 nm for **NBD 2** indicate clean photoconversion with no degradation. The corresponding QC absorption maxima were observed at 237 nm (**QC 1**) and 235 nm (**QC 2**).

**Table 1:** Absorption onsets, absorption maxima, molar extinction coefficients, and quantum yields of conversion to the corresponding quadricyclanes measured in toluene.

Entry	*λ* [nm]	*λ*_onset_ a) [nm]	*ε*_max_ × 10^3^ M^−1^ cm^−1^	Φ [%]
**NBD 1**	308	360	6.0	37
**NBD 2**	311	362	7.9	24

a) Absorption onset is defined as log *ε* = 2.

**Table 2:** Thermodynamic parameters for the thermal reversion of quadricyclanes to norbornadienes in toluene.

Entry	Δ*H* [kJ mol^−1^]	Δ*S* [J K^−1^ mol^−1^]	*t*_1/2_ at 25 °C [days]
**QC 1** to **NBD 1**	110.77	–6.72	76
**QC 2** to **NBD 2**	121.22	–20.10	205

The solar spectrum match is similar for the two compounds; however, the molar extinction coefficient is higher for **NBD 2** than for **NBD 1** (Figure S11, Supporting Information).

**Figure 4:**
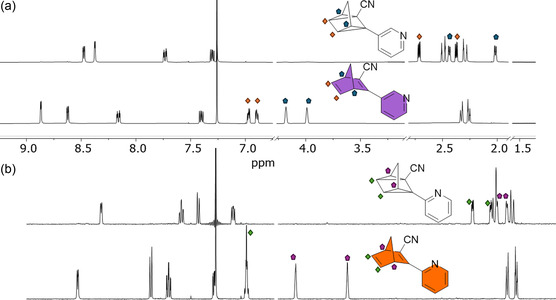
a) ^1^H NMR of **NBD 1** (bottom) and **QC 1** (top) recorded in CDCl_3_; b) ^1^H NMR of **NBD 2** (bottom) and **QC 2** (top) recorded in CDCl_3_.

Nuclear magnetic ressonance (NMR)‐concentration photoconversion (**Figure** **4** and Figure S1–S6, Supporting Information) was investigated in chloroform‐*d* as it is a standard deuterated solvent for conversion studies, and its slightly acidic nature allowed us to assess any spontaneous protonation or decomposition under ambient conditions, offering insight into the intrinsic stability of the compounds. Both **NBD 1** and **NBD 2** could be fully converted to their QC forms after 1 h of irradiation with a 340 nm LED. This is shown through the characteristic loss of the olefinic proton signals (orange diamonds for **NBD 1** and the coalesced peak for **NBD 2**, green diamond, Figure 4), and the appearance of the peaks corresponding to the CH_2_ protons of the QC photoisomers. The upfield shift of the *sp* ^*3*^ methine protons (blue for **QC 1** and purple for **QC 2**) confirms a shielding effect in quadricyclane.

The quantum yields of photoisomerization, Φ_i_, were measured upon irradiation at 310 nm in acetonitrile, to give values of 37% and 24% for **NBD 1** and **NBD 2**, respectively (Table 1and Table S1, Supporting Information). A comparison of similar NBD/QC systems is presented in Table S2, Supporting Information.^[^ ^11^, ^19^, ^24^ ^]^ The yields underscore the influence of substituent effects on photochemical efficiency, offering valuable insights for optimizing MOST systems. Solar conversion efficiencies (Figure S15, Supporting Information) were also calculated and found to be 0.016% and 0.035% for **NBD 1** and **NBD 2**, respectively. These were calculated based on the UV–Vis spectra (Figure S11, Supporting Information), QYs (Table 1), and the energy storage densities (**Table** **3**). The efficiencies are low due to absorption in the UV region of the spectrum.

**Table 3:** Energy storage densities for **QC 1** and **QC 2**, determined by DSC.

Entry	Δ*H* [kJ mol^−1^]	ΔH [kJ kg^−1^]	Δ*H* [kcal mol^−1^]
**QC 1** to **NBD 1**	31.48	162.06	7.52
**QC 2** to **NBD 2**	76.37	393.20	18.25

### 2.3 Kinetic Studies

The kinetics of the thermal reversion of QC to‐NBD were measured at three different temperatures (benchmarked in toluene due to its high boiling point, which supports thermal studies over a broader temperature range) to generate Eyring and Arrhenius plots for all NBD‐QC pairs (Figure S12–S14, Supporting Information). From these, the thermodynamic parameters Δ*H*, Δ*S*, and Δ*G*, along with the rate constant, were determined, allowing extrapolation of the half‐life at room temperature (Table 2). **QC 1** and **QC 2** exhibited significantly longer half‐lives of 75 and 205 days, respectively, compared to previously reported acceptor‐acceptor systems, which range from 5 to 36 h.^[^ ^15^, ^24^ ^]^ Despite their structural similarity, the increased stability of **QC 2** (*ortho*‐substituted) highlights the pronounced influence of pyridine positioning on thermal half‐life. The prolongation of the half‐life of **NBD 2** relative to **NBD 1** is attributed to the higher activation entropy (Δ*S*) and activation enthalpy (Δ*H*) barriers for the QC‐to‐NBD back‐conversion for the former (Table 2). A similar increase in half‐life was previously reported upon *ortho*‐substitution with bulky groups that hinder rotation in the NBD form and hence increase the barrier for the thermal back‐reaction. In the present case, although there is no bulky substituent, the nitrogen in the *ortho*‐position of the pyridine may exert a comparable effect, possibly arising from electronic or conformational influences.^[^ ^11^, ^27^ ^]^

### 2.4 Multimode Switching

**Figure 5:**
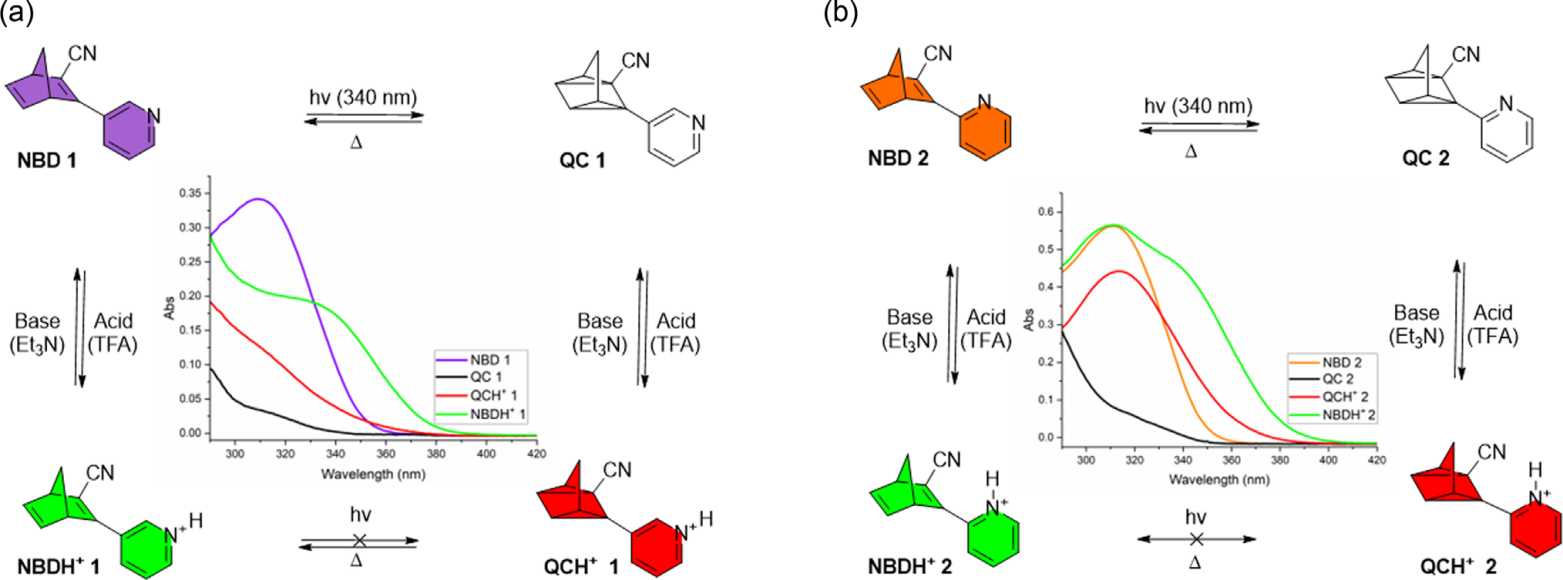
Interconversion scheme of the four molecular species NBD, QC, QCH^+^, and NBDH^+^ in response to light, acid, base, and heat recorded in toluene (toluene is used because it has a higher boiling point and allows direct comparison to literature values of most other NBD systems): a) **NBD 1** (purple curve) undergoes photoconversion to **QC 1** (black curve) upon irradiation with a 340 nm LED. Protonation of **QC 1** with trifluoroacetic acid (TFA, 5 eq.) yields **QCH** ^**+**^ **1** (red curve). Upon heating **QCH** ^**+**^ **1**, it thermally isomerizes back to **NBDH** ^**+**^ **1** (green curve), which can be deprotonated using triethylamine (Et_3_N, 5 eq.) to generate **NBD 1**. A clockwise pattern generates all four species; b) **NBD 2** (orange curve) undergoes photoconversion to **QC 2** (black curve) upon irradiation with a 340 nm LED. Protonation of **QC 2** with trifluoroacetic acid (TFA, 5 eq.) yields **QCH** ^**+**^ **2** (red curve). Upon heating **QCH** ^**+**^ **2**, it does not thermally isomerize back to **NBDH** ^**+**^ **2**, instead, it is locked in the **QCH** ^**+**^ **2** state, and the energy can be stored indefinitely. To generate **NBDH** ^**+**^ **2** (green curve), **NBD 2** was protonated using trifluoroacetic acid (TFA, 5 eq.). A clockwise/anticlock wise pattern does not generate all four species.

The pyridine functionalization of these NBDs allows the observation of multimode switching upon protonation; the interconversion between four distinct molecular species, namely NBD, QC, NBDH^+^, and QCH^+^, has been studied (**Figure** **5** and Figure S7–S10, Supporting Information). Irradiation of **NBD 1/2** at 340 nm induced complete photoisomerization to the corresponding quadricylcanes, **QC 1/2**, while heating the samples enabled full thermal‐reversion to the starting NBDs. Both the NBD and QC forms can be protonated upon the addition of trifluoroacetic acid (TFA, 5 eq.) to generate **NBDH** ^**+**^ **1/2** and **QCH** ^**+**^ **1/2**. As previously noted, protonation causes a bathochromic shift;^[^ ^21^ ^]^ the absorption spectra of the protonated forms NBDH^+^ and QCH^+^ are red‐shifted compared to the corresponding neutral species (Figure 5). The absorption onset of **NBDH** ^**+**^ **1** is ≈390 nm, while for **NBDH** ^**+**^ **2** it extends to ≈400 nm, corresponding to a red‐shift of 30 and 38 nm from the neutral species for **NBD 1** and **NBD 2**, respectively. Despite only a 1 g mol^−1^ difference between phenyl‐substituted norbornadiene^[^ ^19^ ^]^ and **NBD 1/2**, protonation of the latter extends the onset absorption to 400 nm (compared to 358 nm for the former) without compromising molecular weight. Treatment of NBDH^+^ and QCH^+^ with a base (Et_3_N, 5 eq.) regenerates NBD and QC, respectively.

A key distinction between the two photoswitches lies in the half‐life (Table 2) and QCH^+^ state. **QCH** ^**+**^ **1** converts back to **NBDH** ^**+**^ **1** upon heating (Figure 5a and Figure S14, Supporting Information). A decrease in the half‐life of the protonated species was observed in a pyridine‐functionalized DHA/VHF system. However, **QCH** ^**+**^ **2** (LOCK state) does not convert to **NBDH** ^**+**^ **2** when subjected to heat (no energy released). Instead, the system can only release energy in a sequentially governed process (Figure 5b): first, deprotonation, followed by thermal treatment. This behavior can be advantageous for MOST devices, as the energy can be stored indefinitely in the **QCH** ^**+**^ **2** state, offering controlled release only upon deprotonation (Figure 5b).

### 2.5 Energy Storage

**Figure 6:**
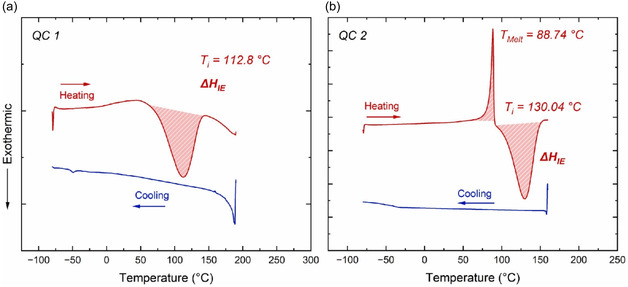
DSC thermogram of **QC 1** a) and **QC 2** b) at a heating/cooling rate of 10 °C under N_2_ atmosphere, showing conversion to the corresponding norbornadienes upon heating and simultaneous heat release.

Differential scanning calorimetry (DSC) analysis provided key insights into the energy storage and heat release properties of the synthesized NBD/QC systems. Measurements at a heating/cooling rate of 10 °C min^−1^ under a nitrogen atmosphere revealed enthalpy changes associated with QC‐to‐NBD backconversion. In both systems, the observed enthalpy changes (Δ*H*_IE_) were primarily attributed to this backconversion, which played a significant role in the overall thermal response. Integration of the broad exothermic peaks yielded Δ*H*_Iso_ values of 162.06 kJ kg^−1^ for **NBD 1** and 393.20 kJ kg^−1^ for **NBD 2**, underscoring their potential for energy storage and controlled thermal energy release within a narrow temperature range (**Figure** **6** **).**

## 3 Conclusion

In summary, we have developed two low molecular weight multimode photoswitches, based on NBD units featuring *meta*‐ and *ortho*‐substituted pyridine functionality (**NBD 1** and **NBD 2**, respectively) and cyano acceptor units, capable of sequential switching between four states (NBD, QC, QCH^+^, and NBDH^+^) in response to light, acid, base, and heat. They represent a class of photoswitches with absorption onset in the ultraviolet range and are studied as a MOST system.^[^ ^16^ ^]^ Both **NBD 1** and **NBD 2** can be quantitatively converted to their higher energy metastable QC forms upon irradiation with UV light, as observed through UV–Vis and NMR studies, with modest quantum yields of isomerization in acetonitrile of 37% and 24%, and theoretical solar conversion efficiencies of 0.016% and 0.035%, respectively. The thermal half‐lives, *t*_1/2_, are 70 and 205 days, respectively, greatly surpassing those of previously reported pyridine‐functionalized NBDs, with the *ortho*‐substituted system showing the highest *t*_1/2_. Upon protonation by the addition of TFA, the absorption spectra are redshifted by up to 30 nm. Notably, **QCH** ^**+**^ **2** functions as a LOCK state (high‐energy isomer), that is capable of storing the captured energy indefinitely. The stored energy can then be released on demand upon addition of a base and thermal reversion back to **NBD 2**. The energy storage densities are 162.06 and 393.2 kJ kg^‐1^ for **NBD 1** and **2**, respectively, with **NBD 2** in particular surpassing the energy storage requirement for a MOST system and falling within the upper range of previously reported acceptor/acceptor‐substituted NBDs. We envision this approach of multimodal photoswitches being applied to future energy storage devices to enable long‐term energy storage (on‐demand controlled release), indirect photosensitization of photoswitches,^[^ ^12^ ^]^ and molecular logic gates.^[^ ^25^ ^]^

## 4 Experimental Section

4.1

4.1.1

##### General Material

All commercial chemicals were used as received. Toluene was dried on an MBraun MB SPS‐800 solvent purification system. Column chromatography was performed on a Biotage Isolera One instrument using pre‐packed silica columns (10 g Biotage SNAP Cartridge). Cyclopentadiene was distilled by cracking dicyclopentadiene over iron filings and stored at −80 °C. Thin‐layer chromatography was carried out using aluminum sheets precoated with silica gel. ^1^H NMR (400 MHz) and ^13^C NMR (101 MHz) spectra were recorded on a Varian 400 MHz instrument, or ^1^H NMR (600 and 800 MHz) and ^13^C NMR (151 MHz) spectra on a Bruker 600 MHz instrument, using the residual solvent as the internal standard (CDCl_3_, ^1^H = 7.26 ppm and ^13^C = 77.16 ppm). All NMR experiments were acquired at 298 K. All chemical shifts are quoted on the δ scale (ppm), and all coupling constants (J) are expressed in Hz. The high‐resolution mass spectra (HRMS) were obtained by WATERS Xevo G2‐XS fitted with QTof or an Agilent 1260 Infinity fitted with an Agilent 6120 quadrupole using ESI mode for ionization. Trifluoroacetic acid (TFA) was used as a source of proton, and triethylamine (Et_3_N)/piperidine was used as a base. All solution‐based spectroscopic measurements were performed in a 1 cm path length cuvette on either a Cary 60 Bio or a Cary 100 UV–Vis spectrophotometer, scanning the wavelength from 700 to 300 nm coupled with Peltier temperature control.

##### NBD 1

To a microwave vial, 3‐(pyridin‐3‐yl) propiolonitrile (225 mg, 1.76 mmol) and cyclopentadiene (696 mg, 10.5 mmol) were dissolved in toluene (3 mL) and sealed. The reaction mixture was heated at 110 °C for 16 h, cooled, and concentrated. The crude mixture was purified by flash chromatography using gradient elution to afford **NBD 1** (130 mg, 38%). *R*_f _= 0.3 (EtOAc/Hexane 8%); ^1^H NMR (400 MHz, CDCl_3_) *δ* = 8.87 (dd, *J* = 2.5, 0.9 Hz, 1H), 8.62 (dd, *J* = 4.8, 1.6 Hz, 1H), 8.16 (ddd, *J* = 8.1, 2.4, 1.6 Hz, 1H), 7.40 (ddd, *J* = 8.1, 4.8, 0.9 Hz, 1 H), 6.97 (dd, *J* = 5.1, 3.0 Hz, 1H), 6.89 (dd, *J* = 5.1, 3.2 Hz, 1H), 4.18 (t, *J* = 2.5 Hz, 1H), 3.99 (t, *J* = 2.4 Hz, 1H), 2.33 (dt, *J* = 7.0, 1.7 Hz, 1H), 2.26 (dt, *J* = 7.0, 1.6 Hz, 1H); ^13^C NMR (101 MHz, CDCl_3_) δ = 167.82, 150.87, 147.22, 143.33, 140.40, 133.73, 129.07, 123.89, 119.95, 117.77, 71.87, 55.22, 53.90; HRMS (ESI+) *m/z* calcd. for C_13_H_10_N_2_ [M + H]^+^: 195.0922; found: 195.0916.

##### NBD 2

To a microwave vial, 3‐(pyridin‐2‐yl) propiolonitrile (125 mg, 0.97 mmol) and cyclopentadiene (386 mg, 5.89 mmol) were dissolved in toluene (2 mL) and sealed. The reaction mixture was heated at 110 °C for 16 h, cooled, and the solvent removed under reduced pressure. The crude was purified by flash chromatography using gradient elution (8% E.A./Hexane) to afford **NBD 2** (100 mg, 53%) as a light yellow solid. ^1^H NMR (400 MHz, CDCl_3_) *δ* = 8.70 (ddd, *J* = 4.8, 1.8, 1.0 Hz, 1H), 7.94 (dt, *J* = 8.0, 1.0 Hz, 1H), 7.75 (td, *J* = 7.8, 1.8 Hz, 1H), 7.30–7.26 (m, 1H), 6.94 (td, *J* = 2.7, 1.0 Hz, 2H), 4.53 (m, *J* = 2.7, 1.4 Hz, 1H), 3.99 (m, *J* = 2.7, 1.5 Hz, 1H), 2.32 (dt, *J* = 7.0, 1.7 Hz, 1H), 2.23 (dt, *J* = 6.9, 1.6 Hz, 1H); ^13^C NMR (101 MHz, CDCl_3_) *δ* = 170.60, 150.97, 149.67, 142.75, 141.64, 137.11, 124.23, 122.45, 117.91, 71.93, 55.70, 53.27; HRMS (ESI^+^) *m/z* calcd. for C_13_H_10_N_2_ [M + H]^+^: 195.0922; found: 195.0915.

## Supporting Information

The authors have cited additional references within the Supporting Information.^[^ ^1^ ^]^

## Conflict of Interest

The authors declare no conflict of interest.

## Supporting information

Supplementary Material

## Data Availability

The data that support the findings of this study are available in the supplementary material of this article.
