# Supplementary material for: Norbornadiene Quadricyclane as Multimode Photoswitches: Synergistic Light and Protonation‐Controlled Heat Release
Source: ChemSusChem. 2025 Aug 19;18(19):e202501005. doi: 10.1002/cssc.202501005 (PMC12487741; doi:10.1002/cssc.202501005)
Supplement: Supplementary file 1 — Supplementary Material [file CSSC-18-e202501005-s001.pdf]

Supporting Information

Norbornadiene Quadricyclane as Multimode Photoswitches: Synergistic Light and Protonation-Controlled Heat Release

Adil S. Aslam[1], Monika Shamsabadi[1], Rebecca J. Salthouse,[4] Joakim Andréasson, and Kasper Moth-Poulsen[1,2,3,4]

1.Department of Chemistry and Chemical Engineering, Chalmers University of Technology, 41296 Gothenburg, Sweden

2.The Institute of Materials Science of Barcelona, ICMAB-CSIC, Bellaterra, 08193 Barcelona, Spain.

3.Catalan Institution for Research & Advanced Studies, ICREA, Pg. Llu´ıs Companys 23, 08010 Barcelona, Spain.

4.Department of Chemical Engineering, Universitat Politècnica de Catalunya, EEBE, Eduard Maristany 10–14, 08019 Barcelona, Spain

Table Of Contents

1) General Materials and Methods 3

2) Experimental Procedures 4

3) NMR Spectra 6

4) Kinetic studies 12

5) Quantum yield calculations 15

6) Solar conversion efficiency 17

7) References 18

1) General Materials and Methods

All reagents were purchased from Fisher or Merck and used as received unless noted otherwise. Toluene was dried on an MBraun MB SPS-800 solvent purification system. Column chromatography was performed on a Biotage Isolera One instrument using pre-packed silica columns (10 g, 25g, or 50g Biotage® SNAP Cartridge). Cyclopentadiene was distilled by cracking dicyclopentadiene over iron filings and stored at -80 °C. Purification of products was carried out by flash chromatography on silica gel (40−63 µm, 60 Å). Thin layer chromatography (TLC) was carried out using aluminum sheets precoated with silica gel. ^{1}H and ^{13}C NMR spectra were recorded on a Varian 400 and a Bruker 800 MHz instrument using the residual solvent as the internal standard ($CDCl_3$, ^{1}H 7.26 ppm and ^{13}C 77.16 ppm). The high-resolution mass spectra (HRMS) were obtained by an Agilent 1260 Infinity fitted with an Agilent 6120 quadrupole using ESI mode for ionization. All UV-Vis absorbance analyses were performed using a Cary 100 UV-Vis. Trifluoroacetic acid (TFA) was used as a source of proton and triethylamine (Et_3N)/piperidine was used as a base. The temperature control during thermal back-conversion was achieved with Peltier temperature control. All photoswitching experiments were performed using a Thorlabs LED with a wavelength of 340 nm. DSC5+ STARe system (METTER TOLEDO) was used to collect the Differential scanning calorimetry (DSC) curves at a heating/cooling rate of 10°C min^{-1} under N_2 atmosphere.

2) Experimental Procedures

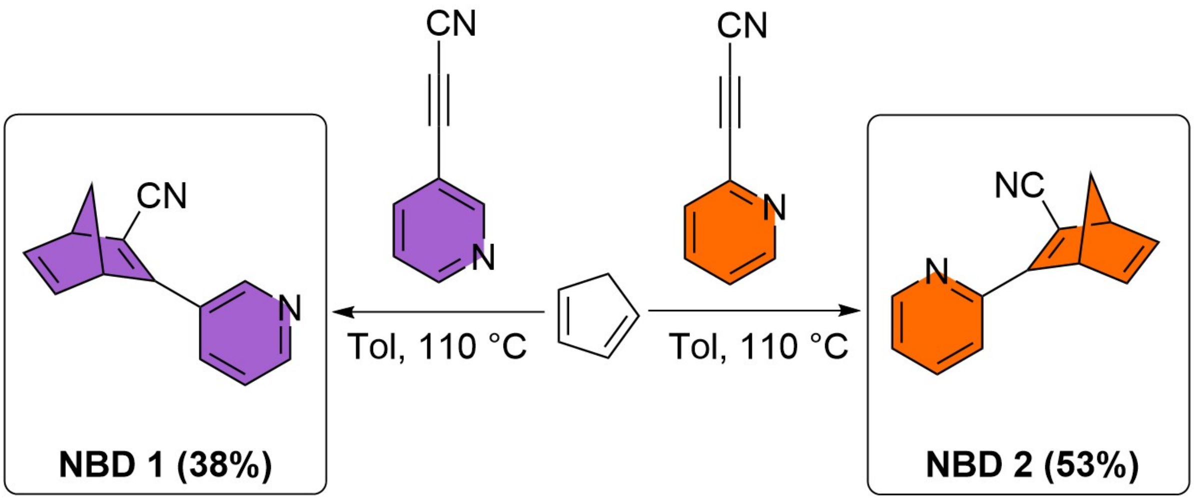

Scheme 1. Synthetic route to NBD 1 and NBD 2 through a Diels-Alder reaction between the respective alkyne and cracked cyclopentadiene.

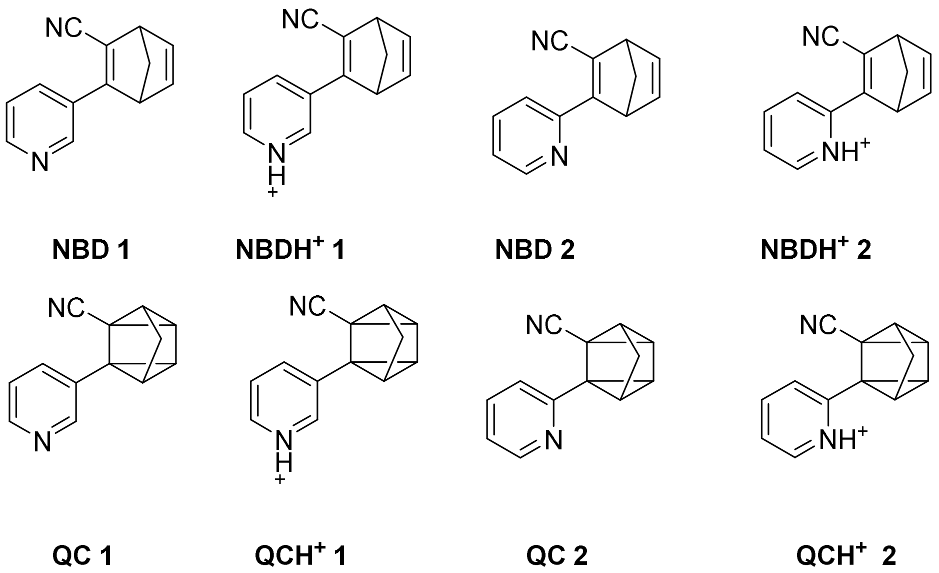

Scheme S2. Structures of NBDs and corresponding QCs

Synthesis of NBD 1

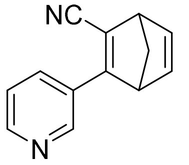

To a microwave vial, 3-(pyridin-3-yl) propiolonitrile (225 mg, 1.757 mmol), and cyclopentadiene (696 mg, 10.54 mmol) were dissolved in toluene (3 mL) and sealed. The reaction mixture was heated at 110°C for 16 hours, cooled, and concentrated. The crude was purified by flash chromatography using gradient elution to afford **NBD 1** (130mg, 38%). R_f= 0.3 (EtOAc/Hexane 8%)**;** ^{1}H NMR (400 MHz, $CDCl_3$) δ= 8.87 (dd, *J* = 2.5, 0.9 Hz, 1H), 8.62 (dd, *J* = 4.8, 1.6 Hz, 1H), 8.16 (ddd, *J* = 8.1, 2.4, 1.6 Hz, 1H), 7.40 (ddd, *J* = 8.1, 4.8, 0.9 Hz, 1H), 6.97 (dd, *J* = 5.1, 3.0 Hz, 1H), 6.89 (dd, *J* = 5.1, 3.2 Hz, 1H), 4.18 (t, *J* = 2.5 Hz, 1H), 3.99 (t, *J* = 2.4 Hz, 1H), 2.33 (dt, *J* = 7.0, 1.7 Hz, 1H), 2.26 (dt, *J* = 7.0, 1.6 Hz, 1H); ^{13}C NMR (101 MHz, $CDCl_3$) δ= 167.82, 150.87, 147.22, 143.33, 140.40, 133.73, 129.07, 123.89, 119.95, 117.77, 71.87, 55.22, 53.90; HRMS (ESI$^+$) *m/z* calc. for $C_{13}H_{10}N_2$ $[M+H]^+$: 195.0922; Found: 195.0916.

Synthesis of NBD 2

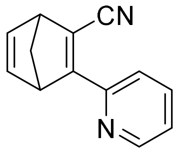

To a microwave vial, 3-(pyridin-2-yl) propiolonitrile (125 mg, 0.97 mmol), and cyclopentadiene (386 mg, 5.89 mmol) were dissolved in toluene (2 mL) and sealed. The reaction mixture was heated at 110°C for 16 hours. The reaction mixture was cooled and concentrated. The crude was purified by flash chromatography using gradient elution (8% E.A./Hexane) to afford **NBD 2** (100mg, 53%) as a solid. ^{1}H NMR (400 MHz, $CDCl_3$) δ= 8.70 (ddd, *J* = 4.8, 1.8, 1.0 Hz, 1H), 7.94 (dt, *J* = 8.0, 1.0 Hz, 1H), 7.75 (td, *J* = 7.8, 1.8 Hz, 1H), 7.30 – 7.26 (m, 1H), 6.94 (td, *J* = 2.7, 1.0 Hz, 2H), 4.53 (m, *J* = 2.7, 1.4 Hz, 1H), 3.99 (m, *J* = 2.7, 1.5 Hz, 1H), 2.32 (dt, *J* = 7.0, 1.7 Hz, 1H), 2.23 (dt, *J* = 6.9, 1.6 Hz, 1H); ^{13}C NMR (101 MHz, $CDCl_3$) δ= 170.60, 150.97, 149.67, 142.75, 141.64, 137.11, 124.23, 122.45, 117.91, 71.93, 55.70, 53.27; HRMS (ESI$^+$) m/z calc. for $C_{13}H_{10}N_2$ $[M+H]^+$: 195.0922; Found: 195.0915.

3) NMR Spectra

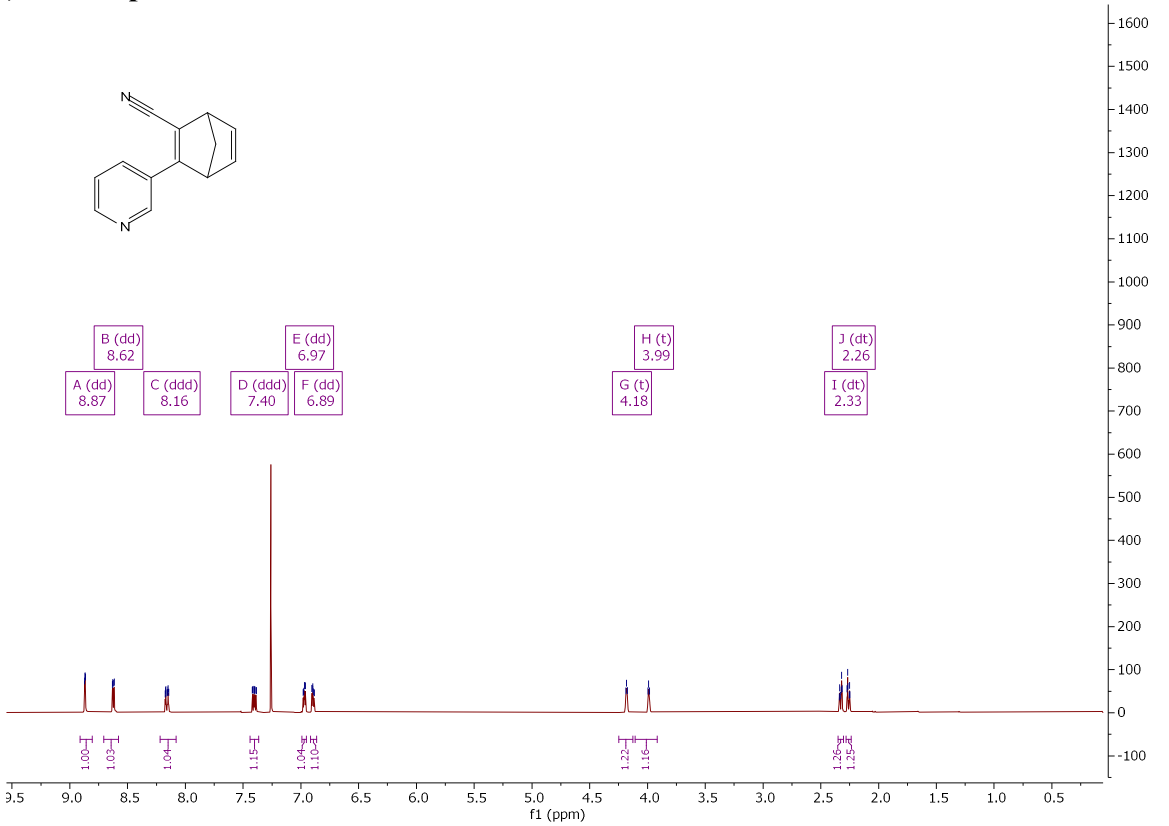

Figure S1 ^{1}H NMR of **NBD 1** recorded in $CDCl_3$

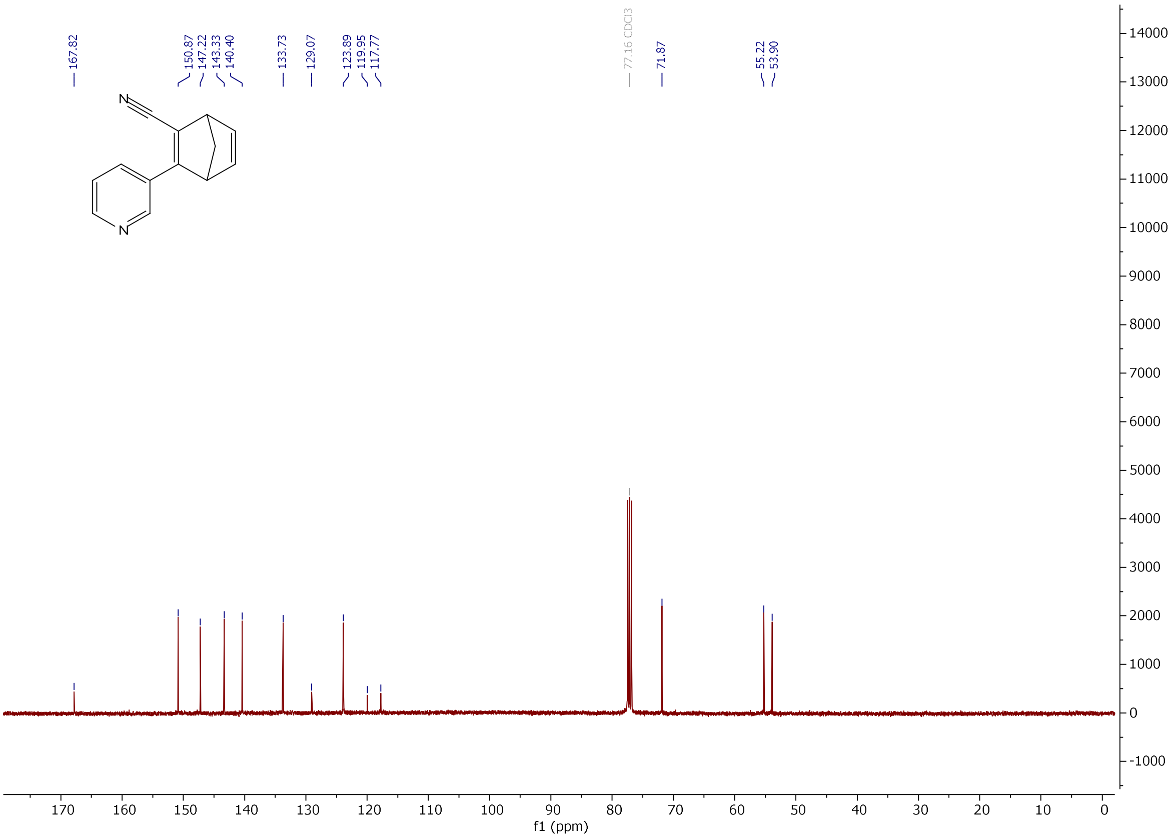

Figure S2 ^{13}C NMR of **NBD 1** recorded in $CDCl_3$

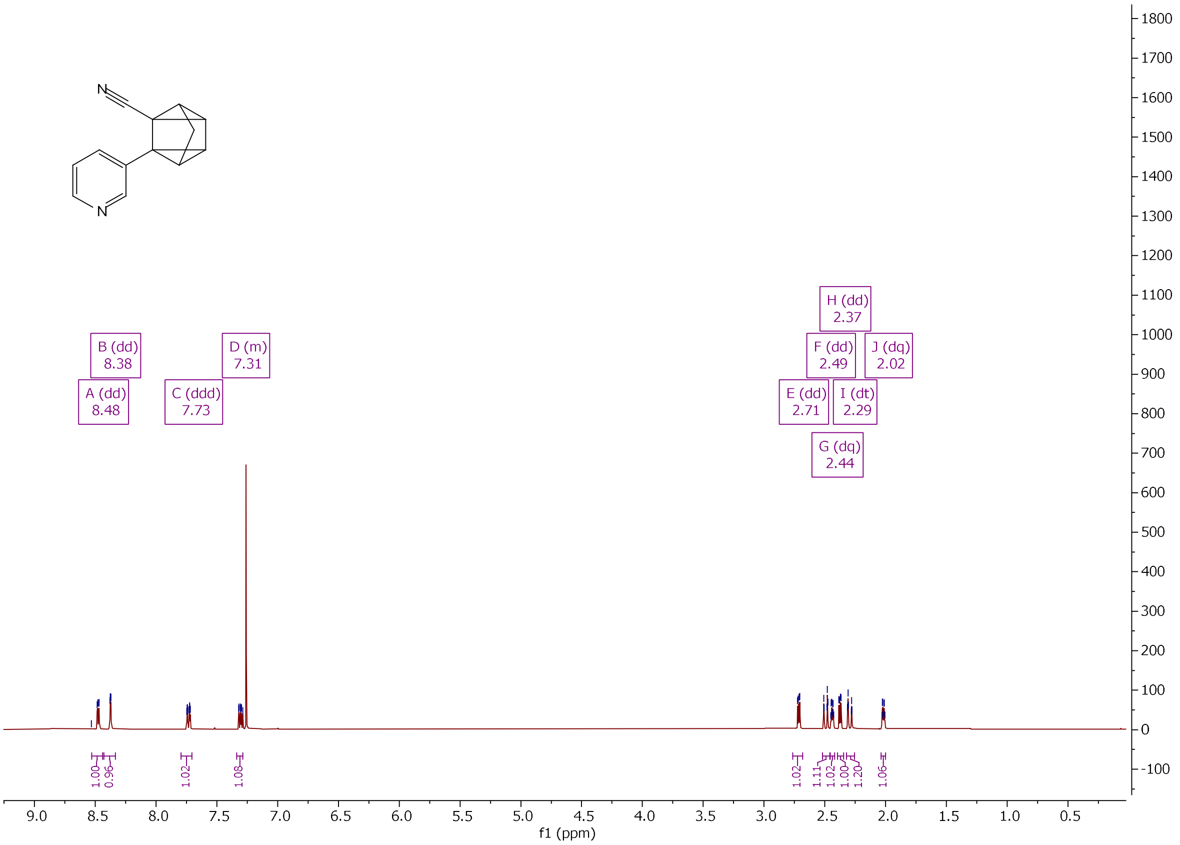

Figure S3 ^{1}H NMR of **QC 1** recorded in $CDCl_3$

QC 1: ^{1}H NMR (400 MHz, $CDCl_3$) δ= 8.48 (dd, *J* = 4.9, 1.6 Hz, 1H), 8.38 (dd, *J* = 2.4, 0.9 Hz, 1H), 7.73 (ddd, *J* = 7.9, 2.4, 1.6 Hz, 1H), 7.34 – 7.29 (m, 1H), 2.71 (dd, *J* = 4.9, 2.6 Hz, 1H), 2.49 (dd, *J* = 12.0, 1.4 Hz, 1H), 2.44 (dq, *J* = 4.9, 1.4 Hz, 1H), 2.37 (dd, *J* = 5.1, 2.6 Hz, 1H), 2.29 (dt, *J* = 12.0, 1.5 Hz, 1H), 2.02 (dq, *J* = 5.1, 1.4 Hz, 1H).

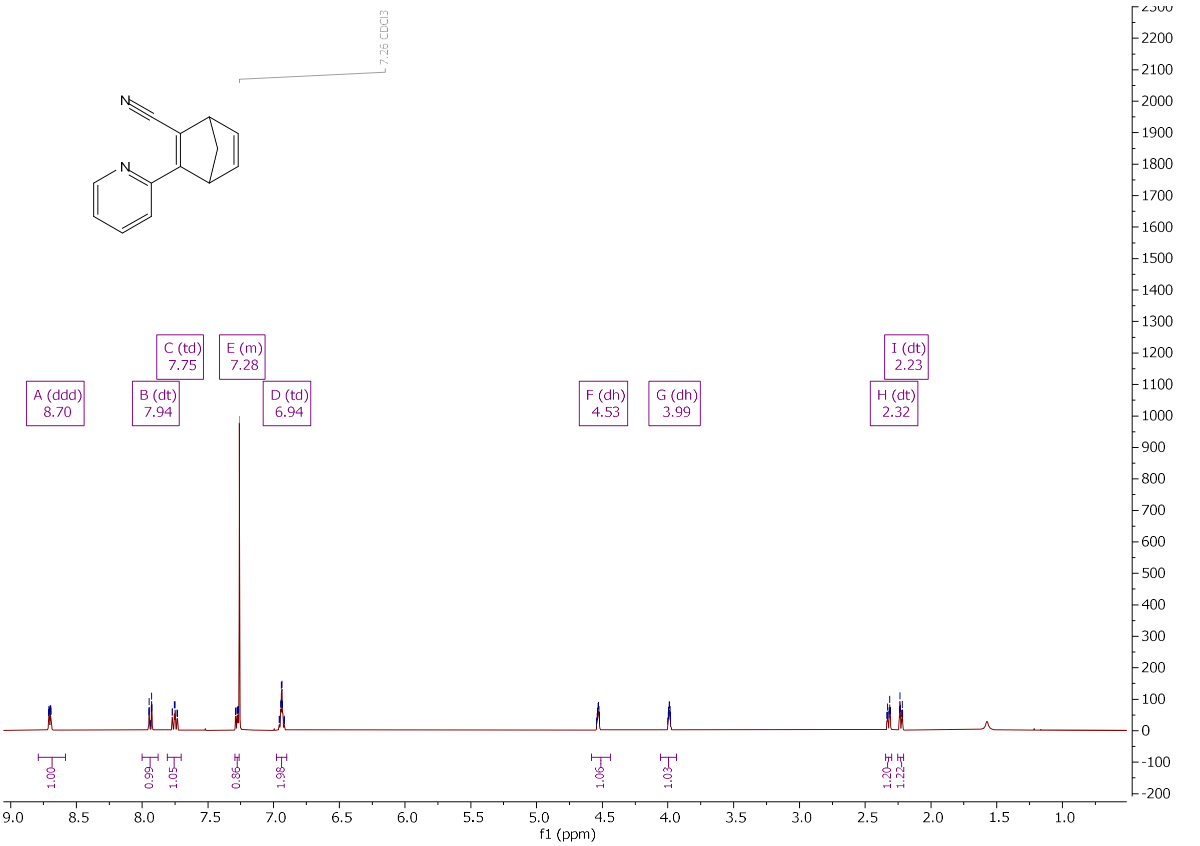

Figure S4 ^{1}H NMR of **NBD 2** recorded in $CDCl_3$

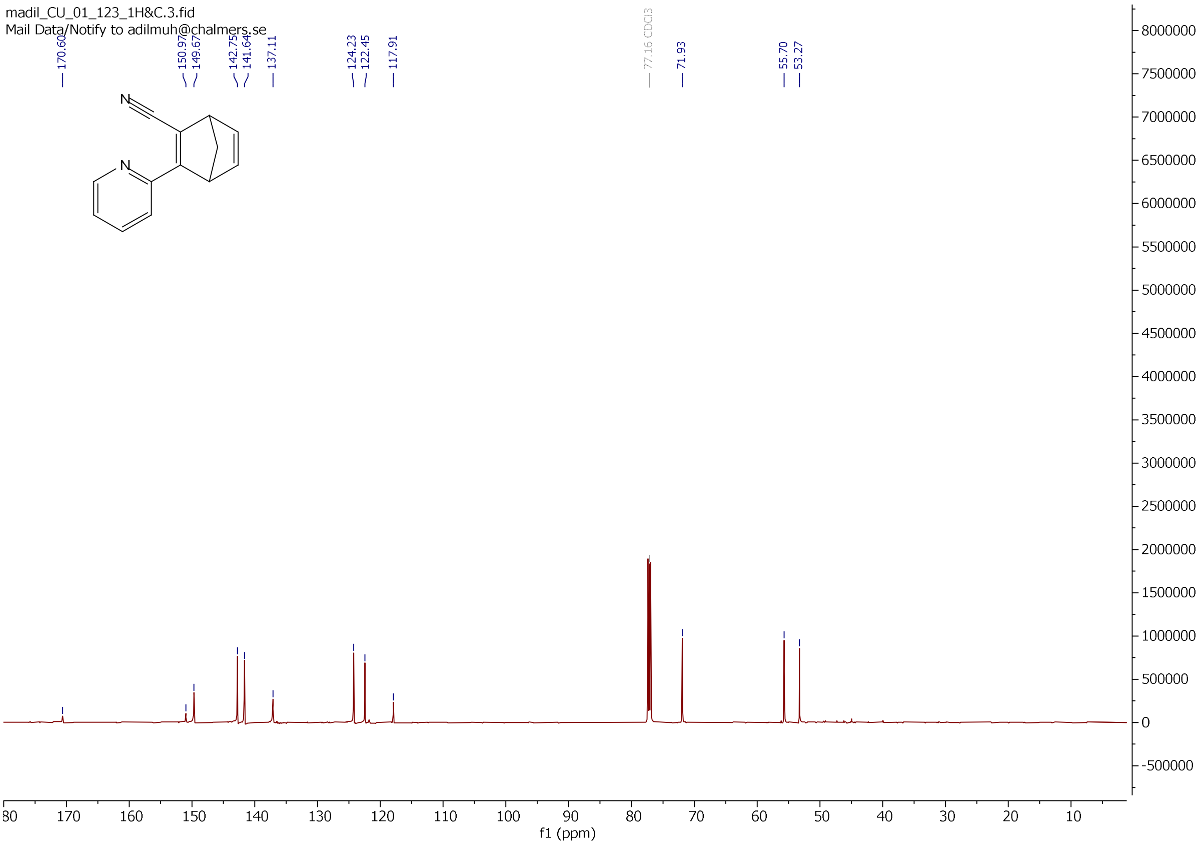

Figure S5 ^{13}C NMR of **NBD 2** recorded in $CDCl_3$

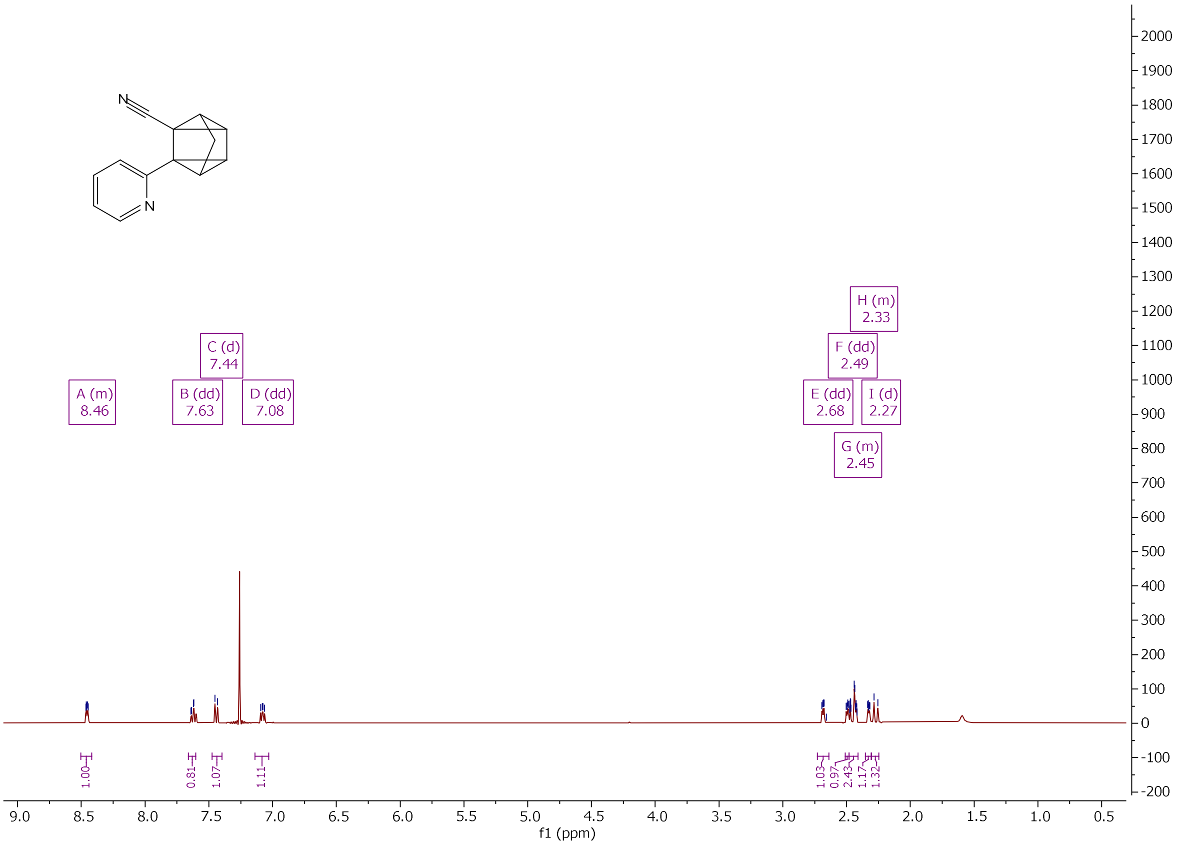

Figure S6 ^{1}H NMR of **QC 2** recorded in $CDCl_3$

QC 2: ^{1}H NMR (400 MHz, $CDCl_3$) δ= 8.50 – 8.42 (m, 1H), 7.63 (dd, *J* = 7.8, 1.8 Hz, 1H), 7.44 (d, *J* = 7.9 Hz, 1H), 7.08 (dd, *J* = 7.6, 4.9 Hz, 1H), 2.68 (dd, *J* = 4.8, 2.5 Hz, 1H), 2.49 (dd, *J* = 5.0, 2.5 Hz, 1H), 2.48 – 2.41 (m, 2H), 2.35 – 2.31 (m, 1H), 2.27 (d, *J* = 11.8 Hz, 1H);

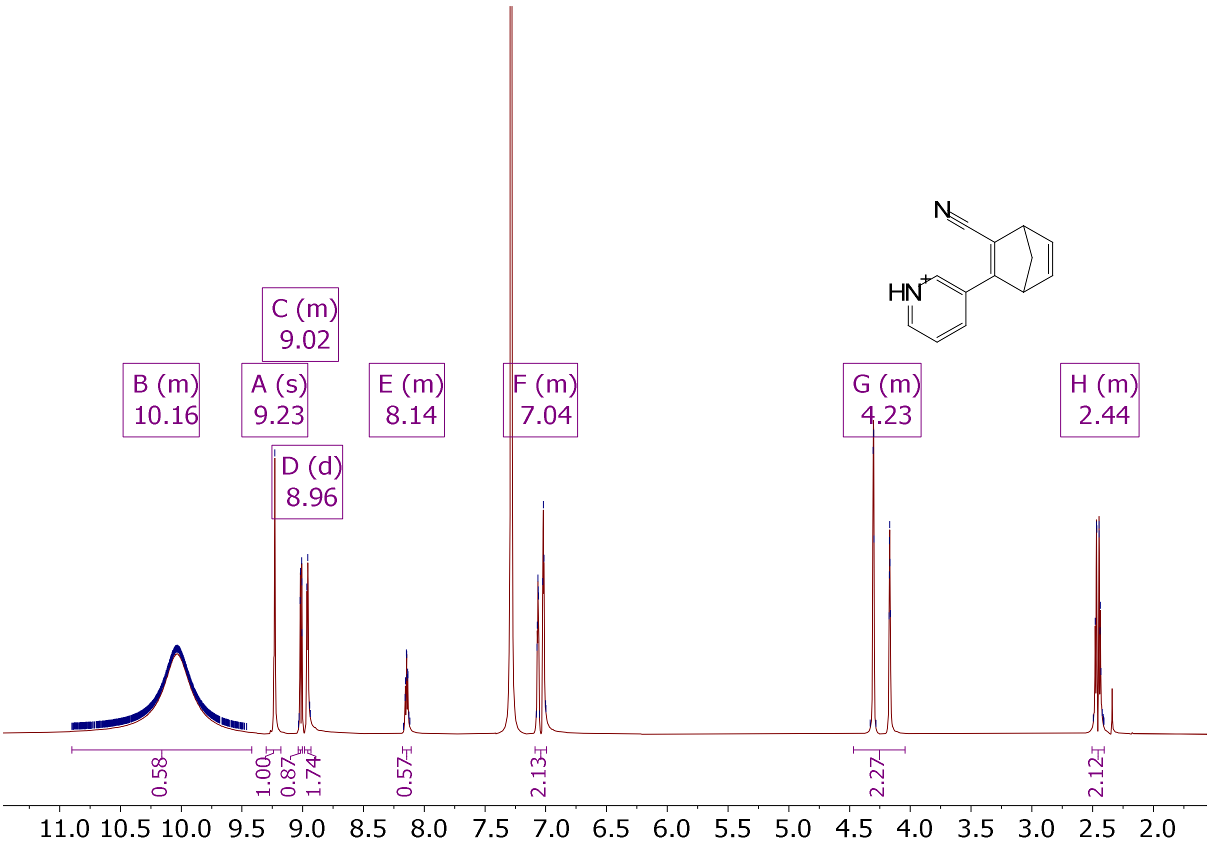

Figure S7 ^{1}H NMR of **NBDH+ 1** recorded in $CDCl_3$

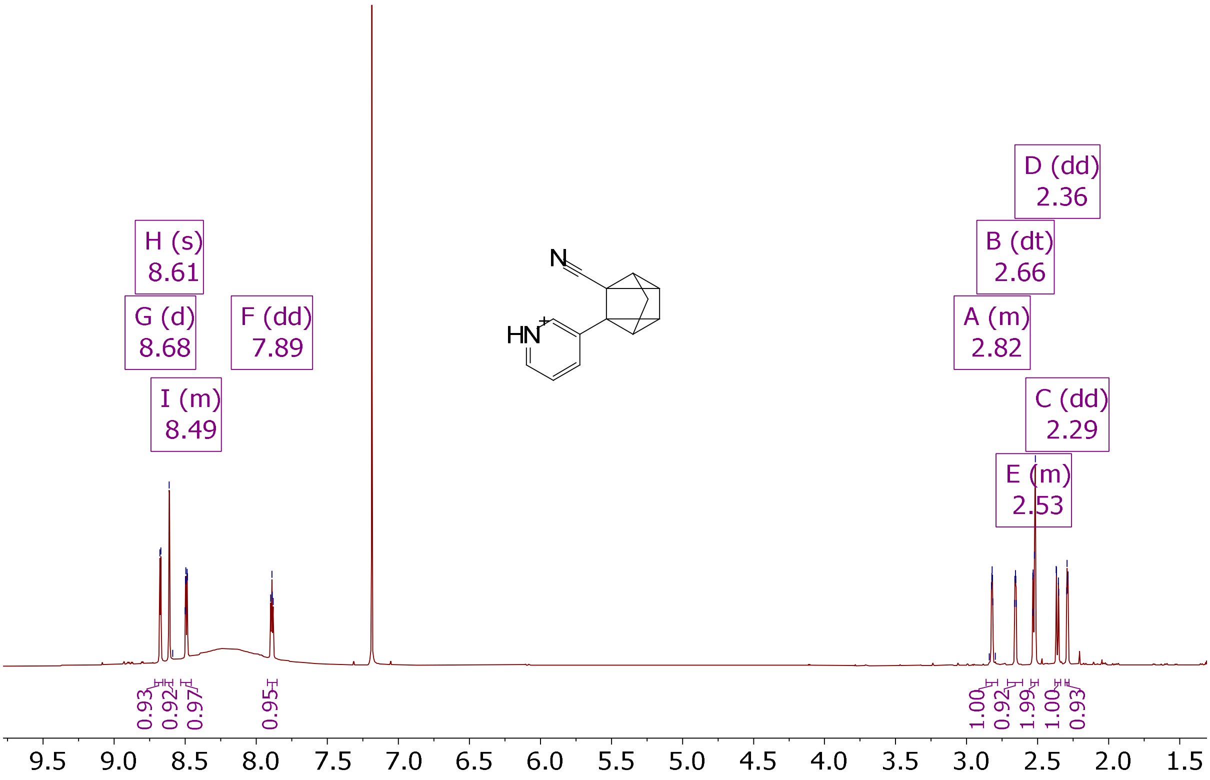

Figure S8 ^{1}H NMR of **QCH+ 1** recorded in $CDCl_3$

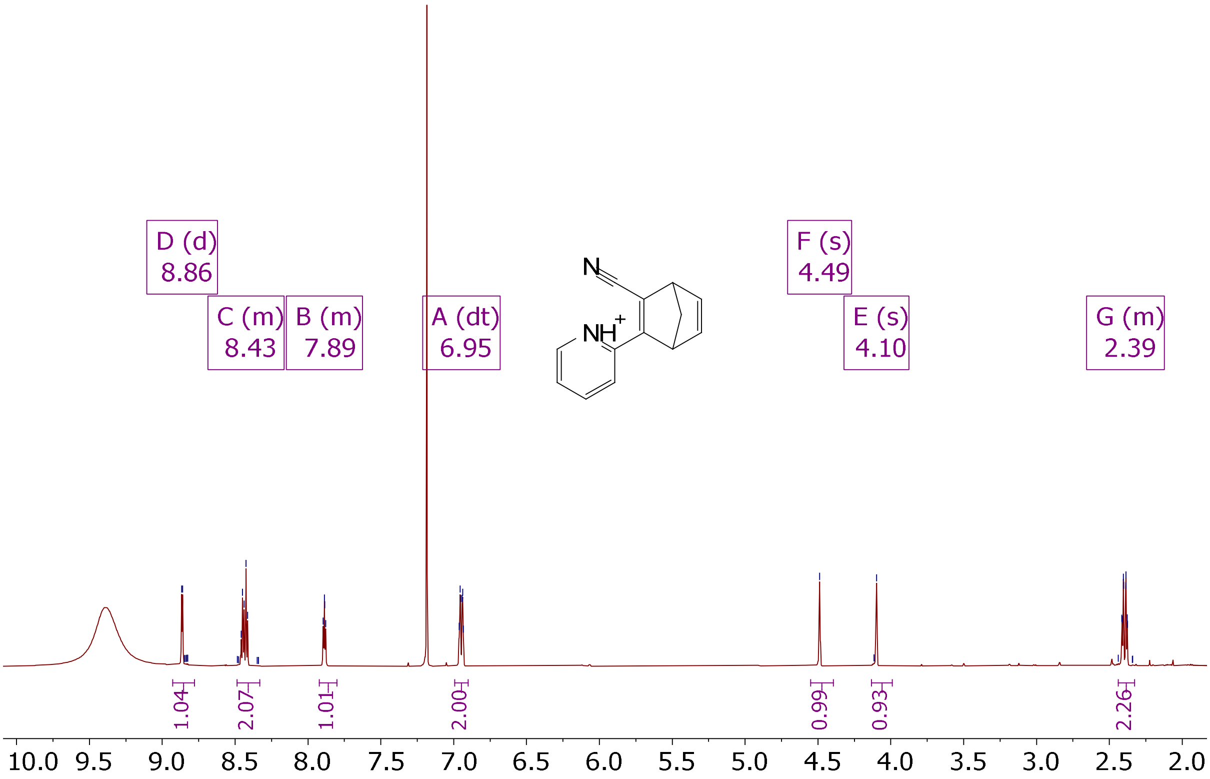

Figure S9 ^{1}H NMR of **NBDH+ 2** recorded in $CDCl_3$

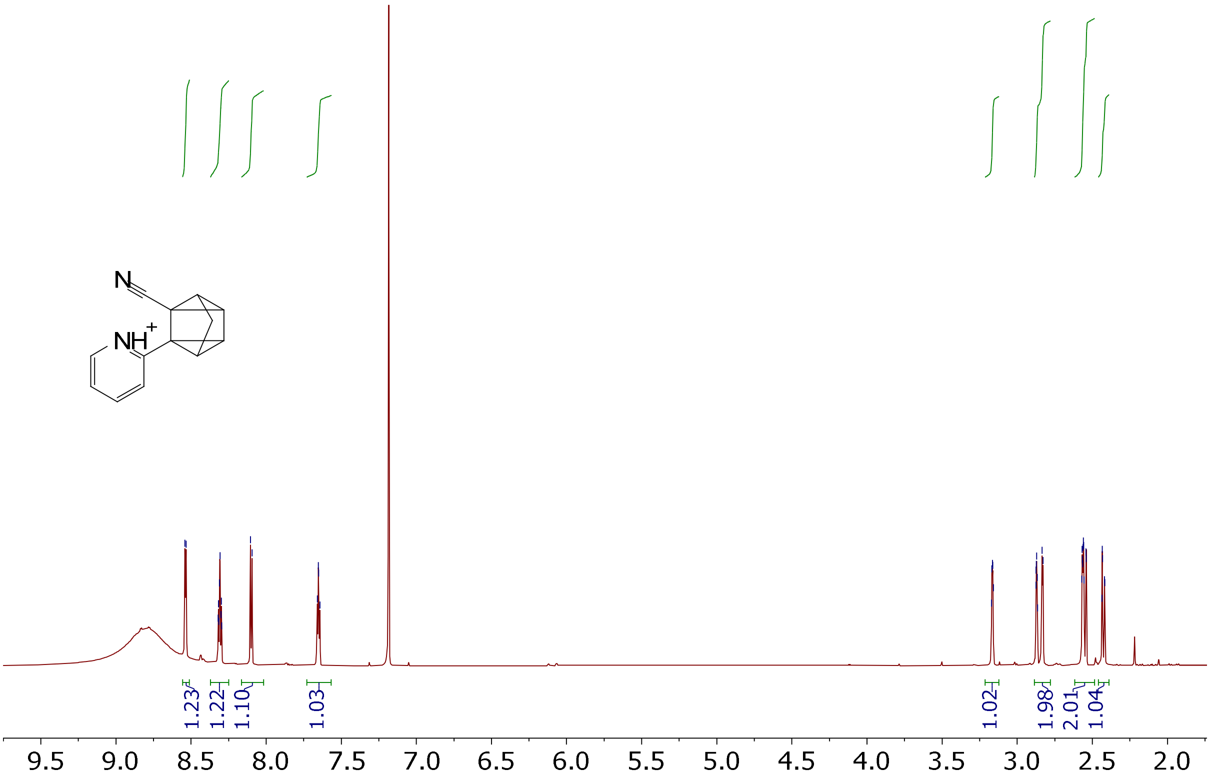

Figure S10 ^{1}H NMR of **QCH+ 2** recorded in $CDCl_3$

4) Kinetic studies

Toluene solutions of NBD derivatives were irradiated until a photostationary state (PSS) was reached in the formation of the corresponding QC isomers, as monitored by UV/Vis spectrophotometry. To study the thermal back-conversion, the solutions were then heated to selected temperatures (333 K, 348 K, 358 K, and 363 K), and the reappearance of the NBD absorption band was monitored over time using a Cary 50 Bio UV/Vis spectrophotometer.

The kinetic traces of each NBD/QC pair were fitted to a monoexponential function to extract first-order rate constants at each temperature. These rate constants were then used to construct Eyring plots (ln(k/T) vs. 1/T), from which the enthalpy ($\Delta H^‡$) and entropy ($\Delta S^‡$) of activation were determined according to the linearized Eyring equation.

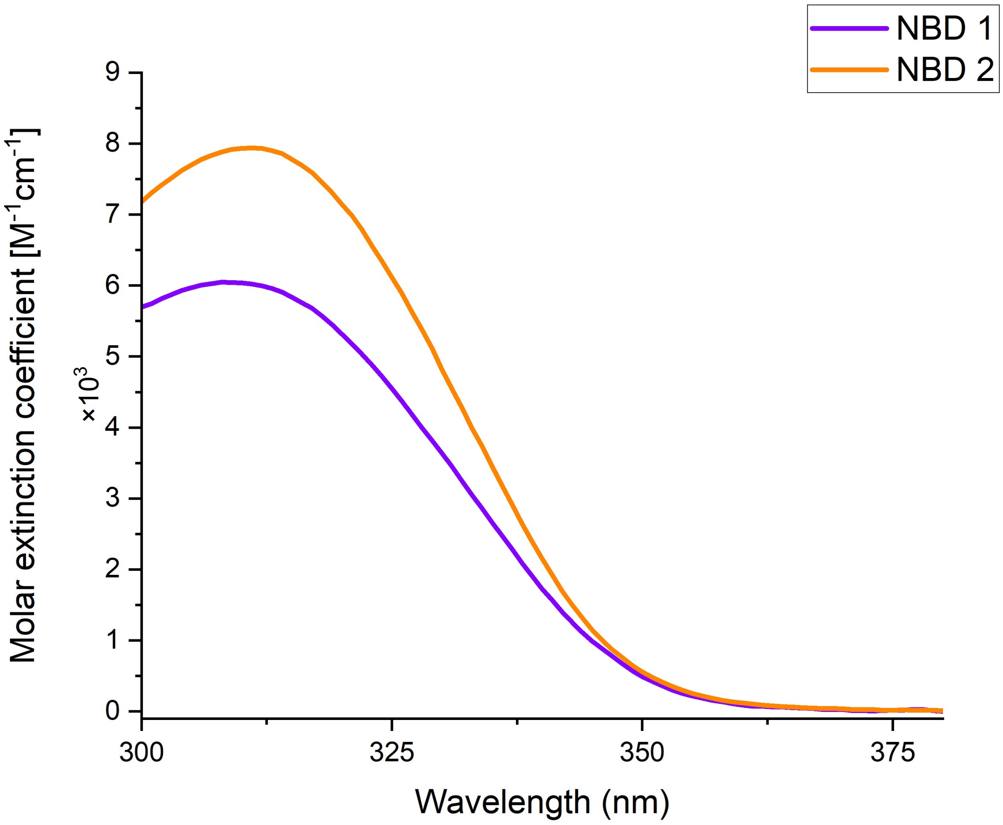

Figure S11 UV absorption profile of **NBD 1** and **NBD 2** recorded in toluene.

QC 1

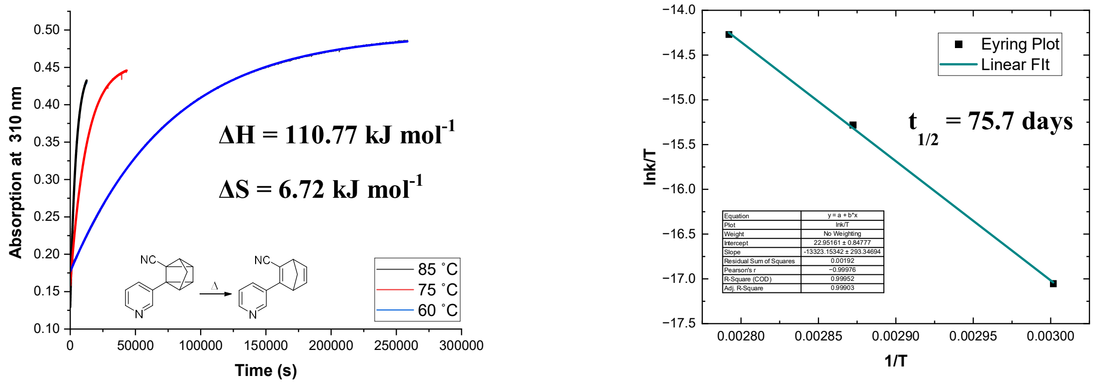

Figure S12 Kinetic study of the back conversion for **QC 1** to **NBD 1** and linear Eyring plot.

QC 2

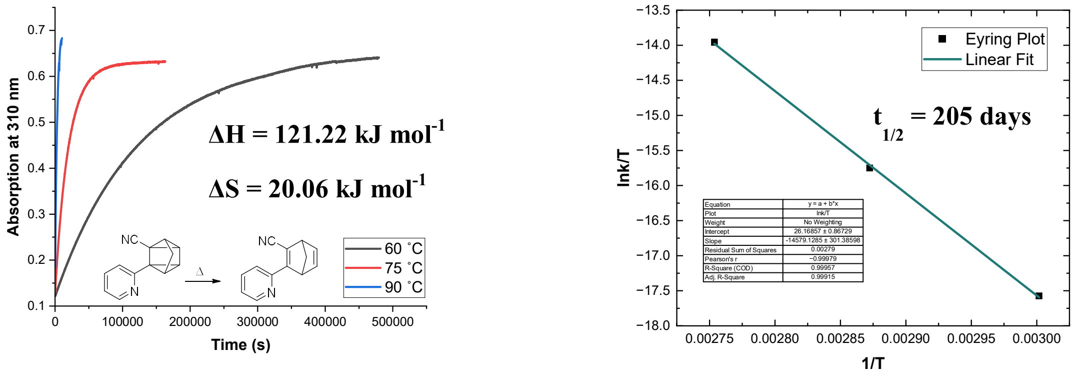

Figure S13 Kinetic study of the back conversion for **QC 2** to **NBD 2** and linear Eyring plot.

QCH+ 1

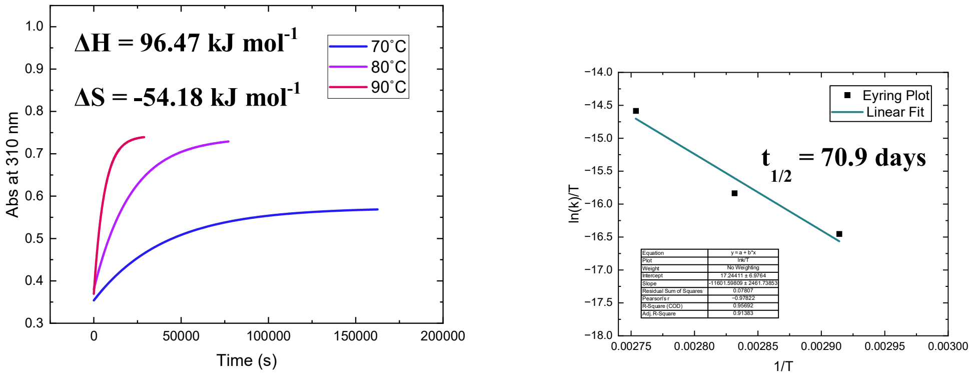

Figure S14 Kinetic study of the back conversion for **QCH$^+$ 1** to **NBDH$^+$ 1** and linear Eyring plot.

5) Quantum yield calculations

The photoisomerization quantum yield was measured following a previously reported method in the high-concentration regime. [1]

Before testing photoisomerization quantum yields, the light source was assumed to be monochromatic with a collimated beam profile. The photon flux of the 310 nm LED lamp was determined using potassium ferrioxalate actinometry. A 3 mL ferrioxalate solution (30 mM in 0.2 N H_2SO_4) was irradiated under stirring. After irradiation, a 0.6 mL aliquot was mixed with 1 mL of buffer (1.2 M NaAc + 0.72 N H_2SO_4) and 2 mL of phenanthroline solution (6 mM). The mixture was then diluted to 25 mL with demineralized water and kept in the dark for 1 hour to allow the reaction to proceed. The photon flux (I, E s^{-1}) was calculated using the following equation:

$$I = slope * \frac{V_1 \cdot V_3}{V_2 \cdot \varepsilon_{510\,nm} \cdot l \cdot \Phi} \tag{S3}$$

where V_1, V_2, and V_3 are the initial solution volume (3 mL), the aliquot volume (0.6 mL), and the final diluted volume (25 mL), respectively. l is the cuvette path length, ε_{510nm} is the molar absorptivity of the tris(phenanthroline) complex (11,100 M^{-1} cm^{-1}), and Φ is the photochemical quantum yield of ferrioxalate decomposition formed from 310 nm irradiation.

To ensure complete photon absorption, solutions were prepared to be optically thick at the wavelength of the irradiation. The absorbance of NBD or QC was continuously monitored over different irradiation times. Once full photon absorption was achieved, the decrease in absorbance followed a linear relationship with irradiation time, described by:

$$\mathrm{A} = A_0 - \frac{\Phi \cdot I}{N_A \cdot V} \cdot t_{irr} \tag{S4}$$

where A and A_0 are the actual and initial absorbance of the NBD/QC solution, N_A is Avogadro's constant, V is the volume of the irradiated sample, and t_{irr} is the irradiation time.

Table SI1 Data from quantum yield measurements

<table>
<tr>
<td>310 nm LED

Photon Flux: 4.47 10^{-9} mol s^{-1}</td>
<td>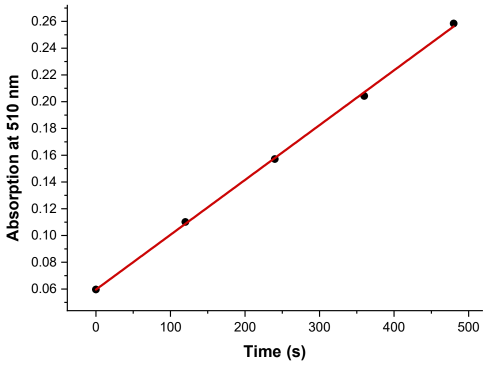
</td>
</tr>
<tr>
<td>NBD 1
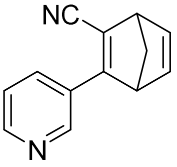

$\Phi_1 = 35\%$
$\Phi_2 = 37\%$
$\Phi_{average} = 37\%$</td>
<td>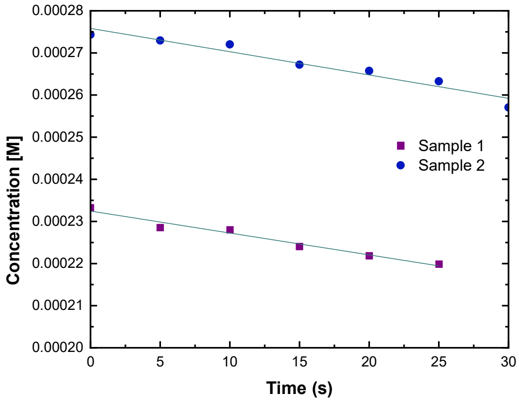
</td>
</tr>
<tr>
<td>NBD 2
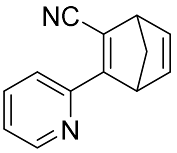

$\Phi_1 = 23\%$
$\Phi_2 = 24\%$
$\Phi_{average} = 23.5\%$</td>
<td>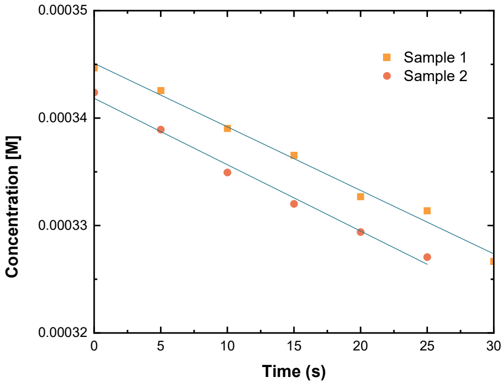
</td>
</tr>
</table>

Table SI2 Comparison of parameters in NBD/QC systems

System	Substituents	λ (nm)	Φ [%]	ΔH (kJ kg^{-1})	$t_{1/2}$ (days)	Ref.
**NBD 1**	m-pyridyl + CN	308	37	162	76	This work
**NBD 2**	o-pyridyl + CN	311	24	393	205	This work
**8**	Pyridine only	355	49		0.25	24
NBD-PhCN	Ph & CN	309	58	629	55	19
NBD-$PhNO_2$	Ph-NO_2 & CN	302	19	318	108	11

6) Solar conversion efficiency

The theoretical solar conversion efficiency (SCE, η_{limit}) limits were calculated using the following equation:

$$\eta_{limit} = \frac{1 \int_0^{\lambda_{onset}} \frac{E_{AM1.5G}(\lambda) \cdot (1 - T(c)) \cdot \phi_{iso} \cdot \Delta H_{storage}}{h\nu \cdot N_A} \cdot d\lambda}{\int E_{AM1.5G}(\lambda) \cdot d\lambda},$$

where $E_{AM1.5G}$ is the solar spectrum measured by Gueymard *et al.*, [2] T(c) is the attenuation, Φ_{iso} is the photoconversion quantum yield, $\Delta H_{storage}$ is the storage energy, $h\nu$ is the photon energy, and N_A is Avogadro's number.

The theoretical solar conversion efficiencies were calculated for **NBD 1** and **NBD 2** using the low concentration UV-Vis spectra in toluene shown in Figure SI11 and the Quantum yields presented in Table 1, and the energy storage densities presented in Table 3.

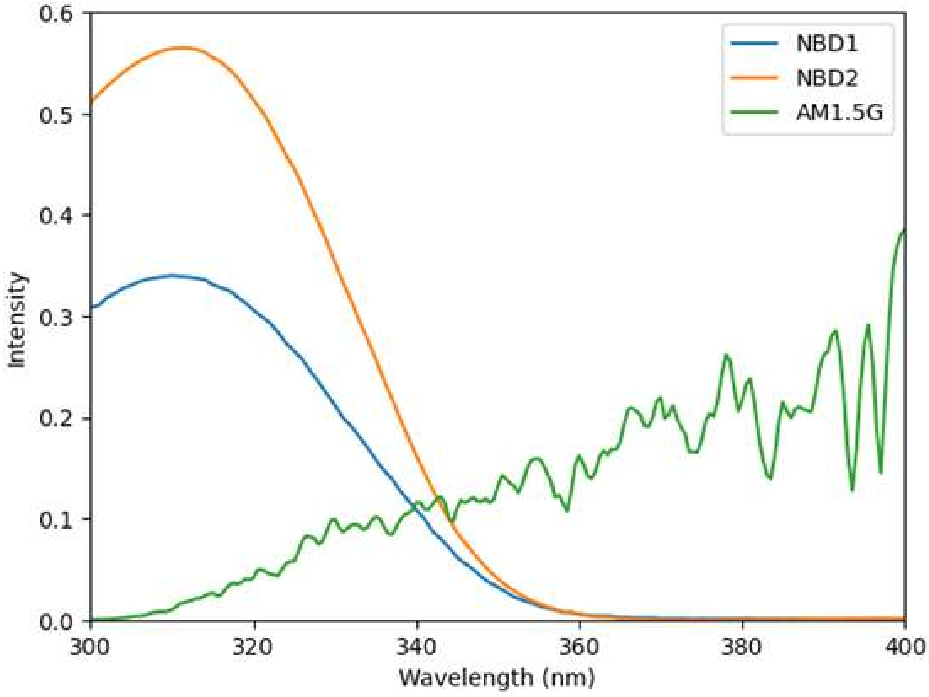

Figure S15 Absorption spectra of **NBD 1** and **NBD 2** at low concentration in toluene and overlap with the AM1.5G solar spectrum used to calculate the solar conversion efficiency limits.

7) References

[1] K. Stranius, K. Börjesson, Sci. Rep. 2017, 7, 41145.

[2] C. A. Gueymard, *Sol. Energy*, 2004, 76, 423–453.
